# Free Vibrations of Bernoulli-Euler Nanobeams with Point Mass Interacting with Heavy Fluid Using Nonlocal Elasticity

**DOI:** 10.3390/nano12152676

**Published:** 2022-08-04

**Authors:** Raffaele Barretta, Marko Čanađija, Francesco Marotti de Sciarra, Ante Skoblar

**Affiliations:** 1 Department of Structures for Engineering and Architecture, University of Naples Federico II, 80138 Naples, Italy; 2 Department of Engineering Mechanics, Faculty of Engineering, University of Rijeka, 51000 Rijeka, Croatia

**Keywords:** fluid-structure interaction, Bernoulli-Euler beam theory, PurelySDM nonlocal model, eigenfrequencies, nanostructures

## Abstract

Eigenfrequencies of a nanobeam with a point mass interacting with a heavy fluid are calculated using Bernoulli-Euler kinematics and consistent nonlocal elasticity model. The proposed approach is applicable to a variety of nanotechnology materials and structures, especially mass nanosensors. Eigenfrequencies are compared with those of local theory and conclusions are drawn. Influence of nonlocal effects, heavy fluid interaction and added point mass on dynamic responses is analyzed and the results are documented. Size phenomena are noted and discussed.

## 1. Introduction

Innovative materials, as carbon nanotubes (CNT) are used in nanosensors, nanoactuators and complex Nano-Electro-Mechanical Systems (NEMS) [1]. A nanofluid is a fluid containing nanometer sized particles, called nanoparticles. From the motion of nanoparticles with the fluid it is possible to define fluid flow in small volumes. Common methodology for microscale fluid flow measurement is cantilever deflection [2,3]. Some nanosensors operate by exploiting eigenfrequencies. For instance, the nanosensor in [4] detects the change in nanotube vibration frequency when a single particle is attached to the carbon nanotube end. The discrepancy between the two frequencies of the nanotubes, with or without the particle attached, is used to measure the mass of the attached particle. Also, as demonstrated in [5,6,7,8], atomic force microscope (AFM) cantilevers often operate in a heavy fluid. This requires calculation of eigenfrequencies of such nanobeam immersed in a heavy fluid. Consequently, fluid-nanostructure interaction modelling, which is the subject of this article, may be of practical interest.

Nowadays, assessment of scale phenomena in nanostructures is effectively achieved using nonlocal and gradient-based continuum formulations of elasticity theory.

Eringen’s nonlocal continuum mechanics differs from classical (local) mechanics in that the stress at a point is defined by the elastic strain at the neighboring points, not just the point under consideration. Specifically, in Eringen’s strain-driven theory, the stress field is convolution integral of the elastic strain field with a suitable averaging kernel [9].

Strain-driven nonlocal theory is however not applicable to bounded nanostructures due to conflicts between constitutive and equilibrium requirements [10,11]. These difficulties have been partially bypassed by resorting to a Two-Phase local/nonlocal strain-driven Model (StrainTPM) adopted in [12], and can be circumvented by resorting to the Purely nonlocal Stress-Driven integral Model (PurelySDM) presented in [13].

The PurelySDM strategy is a special form of Nonlocal Stress Gradient (NStressG) elasticity where gradient length and mixture parameters are zero [14,15] and the theory involves the use of appropriate constitutive boundary conditions. The theory is an example of nonlocal approach that leads to well-posed structural problems in Nano-Mechanics.

In this article, existing results [16] for eigenfrequencies of nanobeams calculated by the PurelySDM nonlocal model are extended to include the influence of a surrounding heavy fluid. Nanobeams are modeled by Bernoulli-Euler theory (BE) [17,18] and all results are compared with classical (local) theory (with and without tip point mass and surrounding heavy fluid-water) [19]. Note that the heavy fluid assumption excludes nanoscale effects on the fluid flow, what requires a significant extension of the presented theory.

## 2. Equilibrium Equations and Boundary Conditions

**Figure 1:**
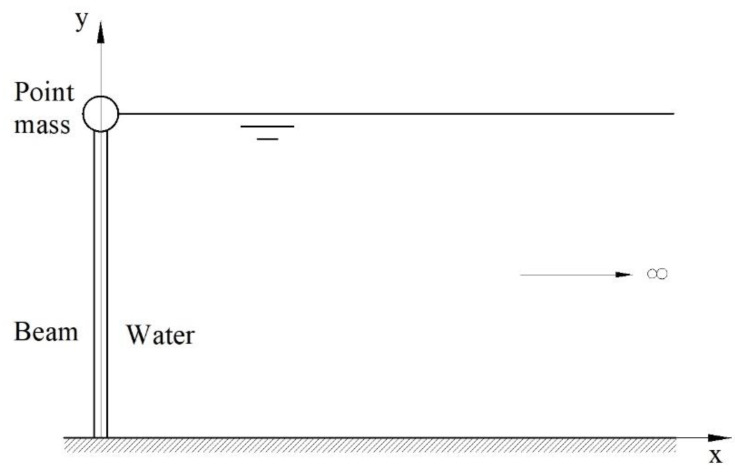
Interaction of cantilever nanobeam with a tip point mass and surrounding heavy fluid.

The kinematics of the plane model of nanobeam vibrations used in this article with a tip point mass immersed in a heavy fluid (water) (Figure 1) is based on BE kinematic assumptions, since the beam is slender. It is also assumed that the beam is initially straight. This is a standard approach in the macroscopic theory of vibrations, where the initial slight curvature of the nanobeam introduced by gravity or fluid pressure is neglected. Further, the influence of the shape and the volume of the tip mass on the surface elasticity of the nanobeam was not considered. This effect could be important for large volumes of the attached nanoparticles. However, their influence on the vibrations is directly included as a point mass with appropriate inertias.

For a nanobeam, defined by the BE theory, the displacement field is expressed by:

(1) $$v=-xu^{\left(1\right)}\left(y,t\right),\;u=u\left(y,t\right)$$

where *v*(*x*,*y*,*t*) is the displacement in the *y*-direction of a point on the cross-section, *u*(*x*,*t*) is the displacement of a point on the centerline (*x* = 0) in the *x*-direction, and *t* is time. The superscript term in parentheses defines the order of the partial derivative with respect to the coordinate *y*.

The axial strain is defined as follows:

(2) $$\varepsilon_{y}=-xu^{\left(2\right)},$$

and the curvature as:

(3) $$\chi \left(y\right)=u^{\left(2\right)}\left(y\right).$$

The equilibrium equation can be defined by the extended Hamilton principle [17]:

(4) $$\int_{t_{1}}^{t_{2}}\left(\delta_{u}T-\delta_{u}V+{\bar{\delta W}}_{nc}\right)dt=0,\;\;\delta_{u}=\delta u\left(y,t\right)={\left.0\right|}_{t=t_{1},t_{2}},\;\;\;\;\;\;\;\;0\leq y\leq L,$$

where *δT* is the variation of the kinetic energy, *δV* is the potential energy of the internal forces, ${\bar{\delta W}}_{nc}$ is the virtual work of the nonconservative forces and *L* is the beam length. For the problem described, the extended Hamilton’s principle has this form

(5) $$\int_{t_{1}}^{t_{2}}\left(\int_{0}^{L}\left(-m\ddot{u}+f\left(y,t\right)-M^{\left(2\right)}\right)\delta udy-{\left.M\delta u^{\left(1\right)}\right|}_{0}^{L}-{\left.J_{0}{\ddot{u}}^{\left(1\right)}\delta u^{\left(1\right)}\right|}_{L}+M^{\left(1\right)}\delta {\left.u\right|}_{0}^{L}-{\left.m_{0}\ddot{u}\delta u\right|}_{L}\right)dt=0,$$

where *m* represents the specific mass with respect to the unit length of the beam, two dots represents the second derivative with respect to time, *f* is the external nonconservative fluid force parallel to the *x*-axis direction, $m_{0}\;$is the mass and $J_{0}$ is the mass moment of inertia of the tip mass at the free end of the beam, and the moment *M* is the moment of internal forces:

(6) $$M\left(y,t\right)=-\int_{A}\sigma_{y}xdA,$$

where *σ*_y_ is the axial stress and *A* is area of the cross-section.

Accounting for arbitrariness of the virtual transverse displacement *δu* in the first term of Equation (5) gives the governing equation:

(7) $$M^{\left(2\right)}-f\left(y,t\right)+m\ddot{u}=0.$$

Taking in arbitrariness of the virtual transverse displacement δ*u* and the virtual rotation $\delta u^{\left(1\right)}$ at the beam ends in Equation (5) provides the boundary conditions as:

(8) $$M^{\left(1\right)}\left(0\right)u\left(0\right)=0,\;\;\;-M\left(0\right)u^{\left(1\right)}\left(0\right)=0,\;\left(M^{\left(1\right)}\left(L\right)-m_{0}\ddot{u}\left(L\right)\right)u\left(L\right)=0,\;\;\;-\left(M\left(L\right)+J_{0}{\ddot{u}}^{\left(1\right)}\left(L\right)\right)u^{\left(1\right)}\left(L\right)=0.$$

The chosen heavy fluid in this analysis is water. Small-amplitude sound waves propagate through water with speed of sound *c* = 1439 m/s [19], indicating that water is considered a compressible fluid since the speed of sound is less than infinity. In addition, the flow is assumed irrotational [20] and the fluid is considered non-viscous, which allows calculation without influence of damping.

Assuming small excitations, the linearized equations describing the specific dynamic pressures *p*(*x*,*y*,*t*) in the heavy fluid are defined as follows:

(9) $$\frac{\partial^{2}p}{\partial x^{2}}+\frac{\partial^{2}p}{\partial y^{2}}=\frac{1}{c^{2}}\frac{\partial^{2}p}{\partial t^{2}},\;0\leq x\leq \infty ,\;0\leq y\leq H=L$$

where *H* is the depth of water which is equal to the length of the beam *L* in this analysis.

The following boundary conditions are used (Figure 1):

-impermeable and rigid bottom of the fluid:

(10) $$y=0\;\;\;\;\to \;\;\;\frac{\partial p}{\partial y}=0,$$

-undisturbed condition at infinite *x* coordinate:

(11) x=∞   →    p=0,

-zero dynamic pressure at the top of the fluid:

(12) y=L=H   →    p=0,

The boundary condition with inclusion of linear free surface waves is defined in Section 2.1.

The interaction between beam and heavy fluid (water) is simplified by having the fluid on one side of the beam. However, the analytical method shown can also be used in situations where the fluid surrounds the beam. In this case, the resultant force is calculated for a ring of points with the same *y*-coordinate [21].

Calculation of interaction in between the beam and fluid is based on expression for correlation of pressure *p* in heavy fluid and acceleration of the beam:

(13) $$x=0,\;\;\;\;\;0\leq y\leq H=L\;\;\;\;\;\;\to \;\;\;\;\frac{\partial p}{\partial x}=-\rho_{f}\frac{\partial^{2}u}{\partial t^{2}},$$

where $\rho_{f}\;$is the fluid density.

The specific pressure *p* describes the action of an external force per unit length of the beam and is therefore equal to the specific force *f*. The final differential equilibrium equation for the beam immersed in the fluid is thus:

(14) $$M^{\left(2\right)}+m\ddot{u}=-p\left(0,y,t\right).$$

### 2.1. Separation of Variables Method

The solution for beam vibrations is assumed, according to the separation of variables method [17], in the form:

(15) $$u\left(y,t\right)=U\left(y\right)T\left(t\right),$$

where *U*(*y*) is the shape of the beam and the function *T*(*t*) is the solution of the equation

(16) $$T^{\prime \prime }+{\hat{\Omega }}^{2}T=0,$$

i.e.,

(17) $$T\left(t\right)=ae^{i\hat{\Omega }t}+be^{-i\hat{\Omega }t},$$

where *a* and *b* are constants, and $\hat{\Omega }$ is the eigenfrequency.

In the case where ${\hat{\Omega }}^{2}<0$, $\hat{\Omega }=i\Omega$, the exponential solution (17) for *T*(*t*) do not represent a natural vibration solution, so only the case ${\hat{\Omega }}^{2}\geq 0$ should be analyzed.

Also using the method of separation of variables, the pressure field in a heavy fluid can be defined as follows:

(18) $$p\left(x,y,t\right)=P\left(x,y\right)T\left(t\right)=X\left(x\right)Y\left(y\right)T\left(t\right),$$

where the product of the functions *X*(*x*) and *Y*(*y*) defines the shape of the pressure field *P*(*x*,*y*).

The function *X*(*x*) is the solution of a differential equation

(19) $$X^{\prime \prime }+{\hat{\alpha }}^{2}X=0,$$

and for exponentially declining wave amplitude through *x* domain has the form:

(20) $$X\left(x\right)=e^{i\hat{\alpha }x},\;\mathrm{or}\;X\left(x\right)=e^{-\alpha x},$$

where $\hat{\alpha }$ is the fluid imaginary wave number, $\hat{\alpha }=i\alpha$, in the *x*-direction [20]. So, for the boundary condition (11), the solution can be easily confirmed, $0=1/\left(\alpha \infty \right)$. It should also be noted, that the boundary condition (11) is satisfied for any real value of α and that there are no nontrivial solutions when ${\hat{\Omega }}^{2}=0$.

The function *Y*(*y*) is the solution of a differential equation:

(21) $$Y^{\prime \prime }+{\hat{\kappa }}^{2}Y=0,$$

and has the form

(22) $$Y=Dcos\left(\hat{\kappa }y\right)+Esin\left(\hat{\kappa }y\right).$$

When the linear free surface condition is not considered, boundary conditions (10) and (12) are used and the solution of Equation (21), i.e., the expression whose zero points define the wave numbers $\hat{\kappa }$ for the *y*-direction of the fluid domain, is:

(23) $$cos\left(\hat{\kappa }H\right)=0,$$

and its solution is

(24) $${\hat{\kappa }}_{n}=\frac{\left(2n-1\right)\pi }{2H},\;\;\;\;\;\;\;\;n=1,2,3,\ldots ,$$

where *n* is the ordinal number of a wave number $\hat{\kappa }$.

If the linear free surface waves are considered, the following boundary condition is used [20]:

(25) $$y=L=H\;\;\;\;\to \;\;\;\;Y^{\prime }-\frac{{\;\hat{\Omega }}^{2}}{g}Y=0,$$

instead of the boundary condition (12), and the solution of Equation (21) is

(26) $$\frac{{\hat{\Omega }}^{2}}{g}+\hat{\kappa }\tan\left(\hat{\kappa }H\right)=0,$$

where *g* is the acceleration of gravity. This expression doesn’t have an analytical solution so the values of the wave number $\hat{\kappa }$ must be solved numerically. From Expression (26), it can be seen that in this case the wave numbers depend on the frequency $\hat{\Omega }$.

Also, the correlation:

(27) $$\alpha_{n}^{2}={\hat{\kappa }}_{n}^{2}-\frac{{\hat{\Omega }}^{2}}{c^{2}}>0,$$

must be fulfilled, because the waves in *x*- and *y*-direction produce a standing wave. Thus, the exact value of the wave number α for any eigenfrequency $\hat{\Omega }$ and wave number $\hat{\kappa }$ can be defined by Equation (27) but the value of α must be real for real values of $\hat{\kappa }$ and $\hat{\Omega }$.

The pressure field (18) is defined by the superposition of the pressure field mode shapes:

(28) $$X\left(x\right)Y\left(y\right)=\sum_{n=n_{1}}^{\infty }G_{n}e^{-\alpha_{n}x}cos\left({\hat{\kappa }}_{n}y\right),$$

where *n*_1_ is the first ordinal number *n* of a wave number $\hat{\kappa }$ for which condition (27) is fulfilled, and *G*_n_ are real constants.

For a more general discussion of the proposed solutions, the following dimensionless parameters are defined:

(29) $$\bar{U}=\frac{U}{L},\;\xi =\frac{y}{L},\;\;{\bar{\kappa }}_{n}={\hat{\kappa }}_{n}L,\;\;\gamma =\frac{\rho_{f}L}{\rho_{s}F}\;\;\mathrm{and}\;\bar{c}=\frac{c}{\Omega_{b}L},$$

where $\bar{U}$, *ξ* and ${\bar{\kappa }}_{n}$ are dimensionless beam mode shape functions, the *y*-coordinate and the wave number in the *y*-direction, respectively; γ is the mass ratio of water to beam, $\bar{c}$ is the dimensionless speed of sound in the fluid, *F* is the beam thickness, and $\Omega_{b}$ is the frequency parameter for the dry beam:

(30) $$\Omega_{b}={\left(\frac{EI_{z}}{\rho_{s}FWL^{4}}\right)}^{1/2},$$

where *E* is the modulus of elasticity of the material of beam, $\rho_{s}\;$ is the density of the beam, *W* is the beam width, and *I*_z_ is the second moment of the cross-sectional area:

(31) $$I_{z}=\int_{A}^{}x^{2}dA,$$

The frequency parameter for a beam immersed in a fluid is:

(32) $$\bar{\omega }\frac{\;\hat{\Omega }}{\Omega_{b}},$$

so the dimensionless wave number for *x*-direction can be calculated using the expression

(33) $${\bar{\alpha }}_{n}^{2}={\bar{\kappa }}_{n}^{2}-\frac{{\bar{\omega }}^{2}}{{\bar{c}}^{2}}.$$

The dimensionless equilibrium equation for the beam is now of the form:

(34) $${\bar{U}}^{\left(4\right)}\left(\xi \right)-{\bar{\omega }}^{2}\bar{U}\left(\xi \right)=-\sum_{n=n_{1}}^{\infty }\underset{A_{n}}{\underbrace{G_{n}e^{-{\bar{\alpha }}_{n}0}}}\cos\left({\bar{\kappa }}_{n}\xi \right),$$

and includes the field of pressures near the beam (*ξ* = 0).

### 2.2. Application of Nonlocal PurelySDM Theory

Since the eigenfrequencies of a beam with nanodimensions are to be calculated, a nonlocal model should be used. In this paper, the nonlocal PurelySDM model is used because it leads to a well-posed structural problem in nanomechanics.

In the present formulation, the integral convolution law of the elastic field is adopted using the stress-driven nonlocal model [13]:

(35) $$\varepsilon_{y}\left(x,y\right)=\int_{0}^{L}\Xi_{\lambda }\left(y-\psi \right)E^{-1}\sigma_{y}\left(\psi ,y\right)d\psi ,$$

where Ξ_λ_ is the special convolution kernel

(36) $$\Xi_{\lambda }\left(y\right)=\frac{1}{2L_{c}}\exp\left(-\frac{\left|y\right|}{L_{c}}\right),$$

*L*_c_ is the characteristic nanobeam length defined by the expression.

(37) $$L_{c}=\lambda L,$$

where *λ* is the dimensionless nonlocal parameter (*λ* > 0).

The integral law by the purely nonlocal stress-driven model [14] is equivalent to the differential problem:

(38) $$\sigma_{y}=-L_{c}^{2}\varepsilon_{y}^{\left(2\right)}E+\varepsilon_{y}E,$$

augmented with a set of constitutive boundary conditions that will be introduced later.

The moment of the internal forces can be defined with the curvature from Equations (2), (4) and (38):

(39) $$M\left(x,t\right)=-L_{c}^{2}EI_{z}\chi^{\left(2\right)}+EI_{z}\chi ,$$

Also, Equation (39) can be reformulated as

(40) $$\chi -L_{c}^{2}\chi^{2}=CM,$$

where *C* = 1/*K* represents the local elastic compliance which is the inverse of the elastic stiffness:

(41) $$K=\int_{A}Ex^{2}dA=EI_{z}.$$

The substitution of the integral convolution law, Equation (35), by the gradient counterpart, Equation (38), is possible only if the constitutive boundary conditions are enforced [11,13,14]:

(42) $$\chi^{\left(1\right)}\left(0,t\right)-\frac{1}{L_{c}}\chi \left(0,t\right)=0,\;\chi^{\left(1\right)}\left(L,t\right)+\frac{1}{L_{c}}\chi \left(L,t\right)=0.$$

After inclusion of the expression (3) into (40):

(43) $$u^{\left(2\right)}-L_{c}^{2}u^{\left(4\right)}=CM,$$

and the second derivative with respect to the dimensionless axial beam coordinate *ξ*, the equation has the form:

(44) $$u^{\left(4\right)}-L_{c}^{2}u^{\left(6\right)}=CM^{\left(2\right)},$$

After substituting Equation (44) into Equation (14), using the dimensionless parameters and some rearrangements, the equilibrium equation for the nanobeam in heavy fluid is thus:

(45) $$\lambda^{2}{\bar{U}}^{\left(6\right)}-{\bar{U}}^{\left(4\right)}+{\bar{\omega }}^{2}\bar{U}=\underset{X\left(0\right)Y\left(\xi \right)}{\underbrace{\sum_{n=n_{1}}^{\infty }A_{n}cos\left({\bar{\kappa }}_{n}\xi \right)}}.$$

For the homogeneous part of Equation (45):

(46) $$\lambda^{2}{\bar{U}}^{\left(6\right)}-{\bar{U}}^{\left(4\right)}+{\bar{\omega }}^{2}\bar{U}=0,$$

an exponential form of the solution is assumed

(47) $$\bar{U}\left(\xi \right)=a\underset{\phi }{\underbrace{e^{\beta \xi }}},$$

which gives the characteristic equation:

(48) $$\lambda^{2}\beta^{6}-\beta^{4}+{\bar{\omega }}^{2}=0.$$

After introducing the parameter $\beta^{2}=\delta +\frac{1}{3\lambda^{2}}$, with the aim to solve the equation with the use of the Cardano formula [22], the new form of the equation is:

(49) $$\delta^{3}+\underset{d}{\underbrace{\left(-\frac{1}{3\lambda^{4}}\right)}}\delta +\underset{f}{\underbrace{\frac{27\lambda^{4}{\bar{\omega }}^{2}-2}{27\lambda^{6}}}}=0,$$

and final expressions for six roots $\beta_{j}$ _j_ of Equation (49) are:

(50) $$\beta_{1}=\sqrt{\delta_{1}+\frac{1}{3\lambda^{2}}},\;\beta_{2}=-\sqrt{\delta_{1}+\frac{1}{3\lambda^{2}}},\;\delta_{1}=S+T,\;\beta_{3}=\sqrt{\delta_{2}+\frac{1}{3\lambda^{2}}},\;\beta_{4}=-\sqrt{\delta_{2}+\frac{1}{3\lambda^{2}}},\;\delta_{2}=-\frac{1}{2}\left(S+T\right)+\frac{1}{2}i\sqrt{3}\left(S-T\right)\;\beta_{5}=\sqrt{\delta_{3}+\frac{1}{3\lambda^{2}}},\;\beta_{6}=-\sqrt{\delta_{3}+\frac{1}{3\lambda^{2}}},\;\delta_{3}=-\frac{1}{2}\left(S+T\right)-\frac{1}{2}i\sqrt{3}\left(S-T\right),$$

where the unknown coefficients *S* and *T* are defined as:

(51) $$S=\sqrt[{3}]{-\frac{f}{2}+\sqrt{D}}\;,\;T=\sqrt[{3}]{-\frac{f}{2}-\sqrt{D}},$$

and the discriminant *D* as:

(52) $$D=\frac{f^{2}}{4}+\frac{d^{3}}{27}.$$

The final solution of the Equation (46) has the form:

(53) $$\bar{U}\left(\xi \right)=\sum_{j=1}^{6}a_{j}\underset{\phi_{j}}{\underbrace{e^{\beta_{j}\xi }}}.$$

The next step is to define a particular solution of Equation (45). Note that the right-hand side of Equation (45) contains the sum of similar elements, indicating that the particular solution also contains a sum of similar elements. So the first step is to find the particular solution for the equation with one such element:

(54) $$\lambda^{2}{\bar{U}}^{\left(6\right)}-{\bar{U}}^{\left(4\right)}+{\bar{\omega }}^{2}\bar{U}=A_{1}cos\left({\bar{\kappa }}_{1}\xi \right),$$

and with the use of method of undetermined coefficients the solution is assumed in the form:

(55) $$\bar{U}=Bcos\left({\bar{\kappa }}_{1}\xi \right)+Csin\left({\bar{\kappa }}_{1}\xi \right),$$

and the solution is:

(56) $${\bar{U}}_{part}=\frac{A_{1}}{-\lambda^{2}{\bar{\kappa }}_{1}^{6}-{\bar{\kappa }}_{1}^{4}+{\bar{\omega }}^{2}}cos\left({\bar{\kappa }}_{1}\xi \right).$$

So, the general solution of Equation (45) has this form:

(57) $$\bar{U}\left(\xi \right)={\bar{U}}_{hom}+{\bar{U}}_{part}=\sum_{j=1}^{6}a_{j}\phi_{j}+\sum_{n=n_{1}}^{\infty }\frac{A_{n}}{{\bar{\omega }}^{2}-\lambda^{2}{\bar{\kappa }}_{n}^{6}-{\bar{\kappa }}_{n}^{4}}cos\left({\bar{\kappa }}_{n}\xi \right).$$

The coefficients *A*_n_ in the particular solution are determined from the fluid-structure interaction condition (13), which, using the definition of fluid pressures (28) and beam displacements (15), has the following form:

(58) $$X^{\prime }\left(x\right)Y\left(y\right)=\sum_{n=n_{1}}^{\infty }G_{n}\left(-\alpha_{n}\right)e^{-\alpha_{n}x}cos\left({\hat{\kappa }}_{n}y\right)=-\rho_{f}\underset{\ddot{u}}{\underbrace{u{\;\hat{\Omega }}^{2}\left(-1\right)}}.$$

After the use of dimensionless variables, the dimensionless expression for the beam-fluid interaction follows:

(59) $$\bar{U}\left(\xi \right)=-\sum_{n=n_{1}}^{\infty }\frac{{\bar{\alpha }}_{n}}{\gamma {\bar{\omega }}^{2}}A_{n}cos\left({\bar{\kappa }}_{n}\xi \right).$$

Equalization of the solution of the equilibrium differential Equation (57) with the equation of the interaction (59) gives:

(60) $$\bar{U}\left(\xi \right)=\sum_{j=1}^{6}a_{j}\phi_{j}+\sum_{n=n_{1}}^{\infty }\underset{B_{n}}{\underbrace{\left(\frac{A_{n}}{{\bar{\omega }}^{2}-\lambda^{2}{\bar{\kappa }}_{n}^{6}-{\bar{\kappa }}_{n}^{4}}\right)}}cos\left({\bar{\kappa }}_{n}\xi \right)=-\sum_{n=n_{1}}^{\infty }\frac{{\bar{\alpha }}_{n}A_{n}}{\gamma {\bar{\omega }}^{2}}cos\left({\bar{\kappa }}_{n}\xi \right),$$

and the new equation is multiplied by $\cos\left({\bar{\kappa }}_{i}\xi \right)$ and integrated with respect to the beam length $\int_{0}^{1}d\xi$ so that the orthogonality of the function *Z* can be used:

(61) $$\sum_{j=1}^{6}\underset{I_{nj}}{D_{j}\underbrace{\int_{0}^{1}cos\left({\bar{\kappa }}_{i}\xi \right)\phi_{j}\left(\xi \right)d\xi }}+\sum_{n=1}^{n_{k}}A_{n}\left(\frac{1}{{\bar{\omega }}^{2}-\lambda^{2}{\bar{\kappa }}_{1}^{6}-{\bar{\kappa }}_{1}^{4}}+\frac{{\bar{\alpha }}_{n}}{\gamma {\bar{\omega }}^{2}}\right)\underset{\int_{0}^{1}Z_{i}\left(\xi \right)Z_{n}\left(\xi \right)d\xi =I_{n}}{\underbrace{\int_{0}^{1}cos\left({\bar{\kappa }}_{i}\xi \right)cos\left({\bar{\kappa }}_{n}\xi \right)d\xi }}=0.$$

The solutions for the integral of orthogonal functions are:

(62) $$\int_{0}^{1}Z_{i}\left(\xi \right)Z_{n}\left(\xi \right)d\xi =\left\{\begin{array}{c} 0,\ldots \;n=i\\ \frac{1}{2}+\frac{sin\left(2{\bar{\kappa }}_{n}1\right)}{4{\bar{\kappa }}_{n}},\;\ldots n>i,i=1,2,3\ldots \end{array}\right\}.$$

In expression (61) *n*_k_ is the number of used pressure field mode shapes. With the growth of *n*_k_ the solution converges.

In the case where the linear free surface waves are not considered, the wave number (24) using dimensionless variables (29) is defined as:

(63) $${\bar{\kappa }}_{n}=\frac{\left(2n-1\right)\pi }{2}=n\pi -\frac{\pi }{2}\;,\;n=1,2,3\ldots ,$$

so final expressions are:

(64) $$\int_{0}^{1}Z_{n}\left(\xi \right)Z_{i}\left(\xi \right)d\xi =\left\{\begin{array}{c} 0,\ldots \;n=i\\ \frac{1}{2}+\underset{0}{\underbrace{\frac{sin\left(\pi \left(2n-1\right)\right)}{2\pi \left(2n-1\right)}}},\;\ldots n>i,i=1,2,3\ldots \end{array}\right\},$$

or if *n*=*i* (*n*=*i*=*1*) the integral has the solution:

(65) $$\int_{0}^{1}cos\left({\bar{\kappa }}_{1}\xi \right)cos\left({\bar{\kappa }}_{1}\xi \right)d\xi =0,$$

while if *i*=1 and *n*>1 the integral has the solution:

(66) $$\int_{0}^{1}cos\left({\bar{\kappa }}_{1}\xi \right)cos\left({\bar{\kappa }}_{n}\xi \right)d\xi =\frac{1}{2}=I_{n}.$$

However, if the linear free surface waves are considered, $I_{n}$ has to be calculated for all ${\bar{\kappa }}_{n}$ using Equation (62).

The final expression for the coefficients $A_{n}$ follows:

(67) $$A_{n}=\underset{E_{n}}{\underbrace{\frac{2}{\frac{1}{\lambda^{2}{\bar{\kappa }}_{n}^{6}+{\bar{\kappa }}_{n}^{4}-{\bar{\omega }}^{2}}-\frac{{\bar{\alpha }}_{n}}{\gamma {\bar{\omega }}^{2}}}}}\sum_{j=1}^{6}a_{j}\underset{I_{nj}}{\underbrace{\int_{0}^{1}cos\left({\bar{\kappa }}_{n}\xi \right)\phi_{j}\left(\xi \right)d\xi }},$$

In the particular solution (56) a new coefficient *B*_n_, and later on ${\tilde{E}}_{n}$, is used:

(68) $$B_{n}=\frac{A_{n}}{-\lambda^{2}{\bar{\kappa }}_{1}^{6}-{\bar{\kappa }}_{1}^{4}+{\bar{\omega }}^{2}}=\underset{{\tilde{E}}_{n}}{\underbrace{\frac{-2}{1-\frac{{\bar{\alpha }}_{n}\left(\lambda^{2}{\bar{\kappa }}_{n}^{6}+{\bar{\kappa }}_{n}^{4}-{\bar{\omega }}^{2}\right)}{\gamma {\bar{\omega }}^{2}}}}}\sum_{j=1}^{6}a_{j}I_{nj},$$

The final general solution of the differential equilibrium Equation (54) has the form:

(69) $$\bar{U}\left(\xi \right)=\sum_{j=1}^{6}a_{j}\phi_{j}+\sum_{n=n_{1}}^{\infty }B_{n}cos\left({\bar{\kappa }}_{n}\xi \right).$$

The classical boundary conditions for a cantilever nanobeam with a tip point mass from (8) are:

(70) $$\bar{U}\left(0\right)=0,$$

(71) $${\bar{U}}^{\left(3\right)}\left(1\right)+\underset{r_{m}}{\underbrace{\frac{m_{0}}{\rho_{s}FL}}}{\bar{\omega }}^{2}\bar{U}\left(1\right)-\lambda^{2}{\bar{U}}^{\left(5\right)}\left(1\right)=0,$$

(72) $${\bar{U}}^{\prime }\left(0\right)=0,$$

(73) $${\bar{U}}^{\left(2\right)}\left(1\right)-\lambda^{2}{\bar{U}}^{\left(4\right)}\left(1\right)=0,$$

where *r*_m_ is the dimensionless ratio of the concentrated mass to the mass of the beam.

Constitutive boundary conditions for the nonlocal PurelySDM method [14] are:

(74) $${\bar{U}}^{\left(3\right)}\left(0\right)-\frac{{\bar{U}}^{\left(2\right)}\left(0\right)}{\lambda }=0,$$

(75) $$-{\bar{U}}^{\left(3\right)}\left(1\right)-\frac{{\bar{U}}^{\left(2\right)}\left(1\right)}{\lambda }=0,$$

Expression (71) contains the boundary condition for a point mass at a free beam end, while rotational inertia effects are neglected.

### 2.3. Definition of the Eigenfrequencies of the Beam-Fluid System

The system of equations with classical and constitutive boundary conditions (70)–(75) applied to the general solution of the differential equilibrium Equation (69) gives six homogeneous algebraic equations with six unknowns *C*_i_ and unknown eigenfrequency $\bar{\omega }$. These algebraic equations can be rewritten in the matrix form as the product of a quadratic matrix of linearly independent equation solutions, *A*($\bar{\omega }$), and the vector of constants *C*_i_, *p*:

(76) $$A\left(\bar{\omega }\right)p=0.$$

If the determinant of an *n* × *n* homogeneous system of equations is zero, then the system has an infinite number of solutions (in this case eigenfrequencies $\bar{\omega }$). Thus, the values of the eigenfrequencies $\bar{\omega }$ are defined as zero points of the determinant of the matrix *A*($\bar{\omega }$).

## 3. Examples

### 3.1. Convergence and the Influence of Nonlocal Parameter

At the beginning of the numerical analysis it is necessary to define the number of pressure field mode shapes (*n*_k_) needed for the convergence of the results. The first part of this example deals with this issue. The beam length is *L* = 100 nm, the thickness *F* = 3.85 nm, the mass density $\rho_{s}$ = 2600 kg/m^3^ and density of fluid is $\rho_{f}$ = 1000 kg/m^3^ (water), so dimensionless parameter mass ratio of water to beam is *γ* = 10. Width of the beam is *W* = 10 nm and modulus of elasticity is *E* = 160 GPa. The linear free surface waves are not considered, i.e., *p*(*x*,*y* = *H*,*t*) = 0. Water is compressible and the speed of sound is *c* = 1439 m/s. The beam is completely immersed in water and there is no tip mass. Solutions in this and other examples were obtained by the aid of Wolfram Mathematica software. Possible numerical ill-conditioning didn’t appear [23].

**Figure 2:**
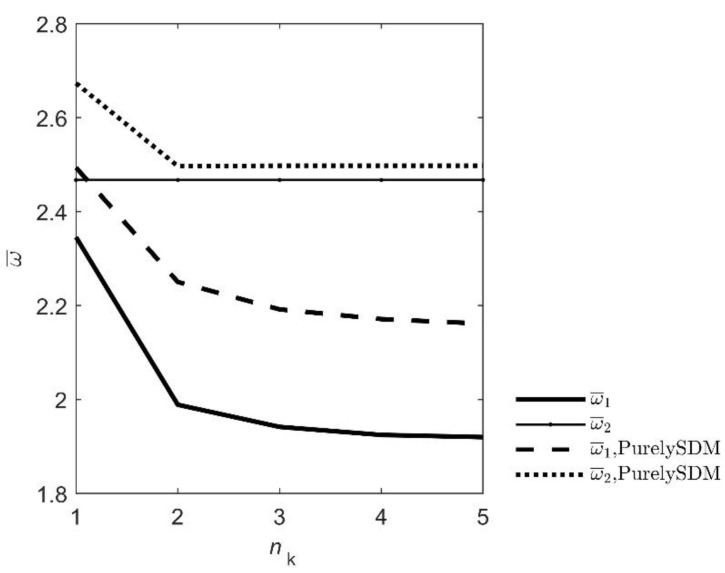
Convergence of eigenfrequencies with the growth of parameter *n*_k_.

From the diagram in Figure 2 and corresponding values in Table 1, it can be seen that the results converge at *n*_k_ = 4, so calculations will be performed with this value. Also, it can be seen that frequency ${\bar{\omega }}_{2}$ calculated with local theory, which is defined as the eigenfrequency of water domain in the rigid beam case [19], do not change its value with growth of *n*_k_. But, it can be also seen, that second frequency calculated with nonlocal theory changes with growth of *n*_k_ which points out that frequencies connected with fluid are influenced by nonlocal theory. The reason for this new effect can be explained with the expression for fluid standing wave (28, 34) where coefficient *G_n_* is now a function of *λ*. If the fluid standing wave changes then its eigenfrequency also changes.

**Figure 3:**
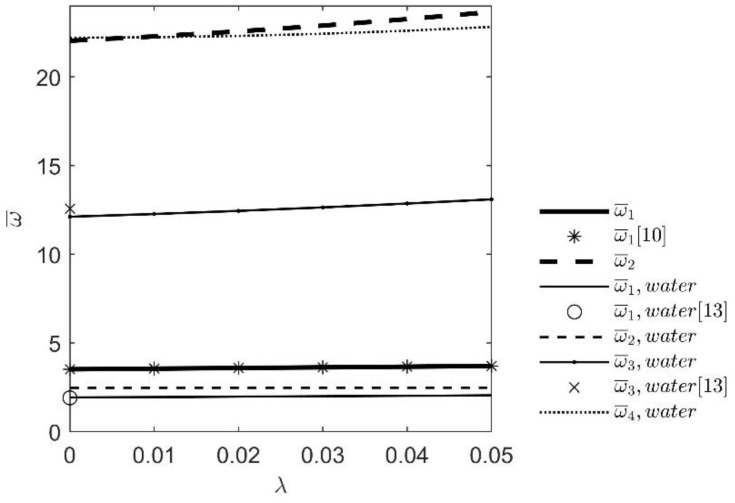
Influence of the dimensionless nonlocal parameter growth on the eigenfrequencies of dry nanobeam and nanobeam immersed in water.

The analysis of the influence of the dimensionless nonlocal parameter (0 ≤ *λ* ≤ 0.06) on eigenfrequencies of defined beam with and without surrounding water follows. The results are shown in Figure 3 and Table 2.

From the given results, it is evident that the nonlocal PurelySDM method for the given boundary conditions causes stiffening effects for nanobeams. The effect is observed for dry beams as well as for beams immersed in water.

### 3.2. The Influence of Tip Point Mass, Water and Nano-Scale Effects

**Figure 4:**
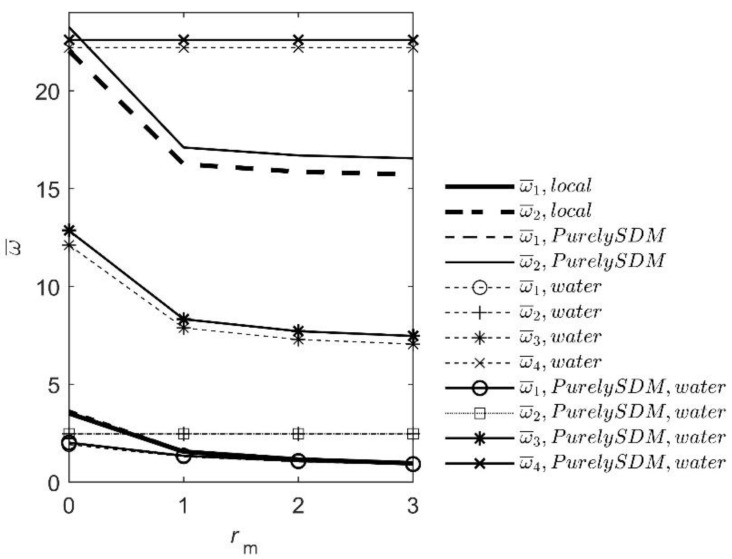
Influence of tip point mass, water and nano-scale effects.

In Example 3.2, the effect of tip point mass on the free end of the dry beam and the beam immersed in water is analyzed using the local and nonlocal theory. The geometry of the beam is the same as in Example 3.1. The only difference is in the value of the dimensionless tip mass *r*_m_ = 0, 1, 2 and 3. The number of used pressure field mode shapes is *n*_k_ = 4. The results are shown in Figure 4 and Table 3.

After comparing the results, it can be seen that the eigenfrequencies decrease with the growth of the tip mass for both the local and nonlocal theories. The difference is that the results for the nonlocal PurelySDM theory are slightly higher because the nanobeam is slightly stiffer. On the other hand, the reduction of the eigenfrequency with the growth of the tip mass decreases when the beam is immersed in water. This effect can have a crucial impact on the functionality of nanosensors.

## 4. Conclusions

Modeling nanoscale effects on a nanobeam is necessary because such events occur in experiments with different types of materials, boundary conditions and morphology of nanostructured materials. In this article, the PurelySDM nonlocal model is used as a nonlocal approach that leads to well-posed structural problems in nanomechanics.

The method was applied to the analysis of the dynamic behavior of the interaction between a beam with a tip point mass and water, with the boundary conditions of zero dynamic pressure at the water surface. The equations defining the system are solved using the method of separation of variables. The equation for eigenfrequencies is derived and the exact values are determined using numerical and analytical approaches.

The analyses in the Examples section lead to the following main conclusions:

1. an increase of the nonlocal parameter of PurelySDM method leads to an increase of the eigenfrequencies for all tested boundary conditions of the system beam-water, indicating a higher stiffness,
2. the increase in point mass leads to a decrease in eigenfrequencies of a nanobeam for both local and nonlocal theory,
3. the calculated eigenfrequencies of the coupled system with the local and nonlocal theory (corresponding to frequencies of the water domain) are equal for various tip point masses, but growth of eigenfrequencies occurs with the growth of the nonlocal parameter of PurelySDM method,
4. when the beam is immersed in water, the main effect of the tip point mass (decrease of the eigenfrequency) is reduced for local and nonlocal theories. This effect can have a crucial impact on functionality of nanosensors.

## Figures and Tables

**Table 1:** Convergence of eigenfrequencies.

*n*_k_	1	2	3	4	5
local theory					
${\bar{\omega }}_{1}$	2.3456	1.9889	1.9418	1.9247	1.9169
${\bar{\omega }}_{2}$	2.4674	2.4674	2.4674	2.4674	2.4674
nonlocal PurelySDM theory, *λ* = 0.1					
${\bar{\omega }}_{1}$	2.4936	2.2502	2.1919	2.1712	2.1615
${\bar{\omega }}_{2}$	2.6732	2.4967	2.4977	2.4977	2.4977

**Table 2:** Influence of the dimensionless nonlocal parameter growth on the eigenfrequencies.

**λ**	**0**	**0.01**	**0.02**	**0.03**	**0.04**	**0.05**
*n_k_* = 0						
${\bar{\omega }}_{1}$	3.5160	3.5515	3.5877	3.6246	3.6621	3.7002
${\bar{\omega }}_{1}$ [10]	3.516013	3.551528	3.587734	3.624609	3.662122	3.700236
${\bar{\omega }}_{2}$	22.0345	22.2764	22.5608	22.8868	23.2523	23.6552
*n_k_* = 4						
${\bar{\omega }}_{1}$	1.9247	1.9482	1.972	1.9961	2.0206	2.0452
${\bar{\omega }}_{1}$ [13] ^1^	1.9047	-	-	-	-	-
${\bar{\omega }}_{2}$	2.4674	2.4677	2.4686	2.4701	2.4723	2.475
${\bar{\omega }}_{3}$	12.1148	12.2681	12.4436	12.6404	12.8574	13.0929
${\bar{\omega }}_{3}$ [13] ^1^	12.5670	-	-	-	-	-
${\bar{\omega }}_{4}$	22.2066	22.2313	22.305	22.4274	22.5977	22.8147

^1^ ${\bar{\omega }}_{1}$ and ${\bar{\omega }}_{2}$ are calculated from dimensionless frequency *ω* in ([13], Table 1 and Table 2) which is correlated to $\bar{\omega }$ with expression $\bar{\omega }=\omega^{2}$.

**Table 3:** Influence of tip point mass, water and nano-scale effects (linear free surface waves not considered).

*r*_m_	0	1	2	3
local theory, no fluid				
${\bar{\omega }}_{1}$	3.5160	1.5573	1.1582	0.9628
${\bar{\omega }}_{2}$	22.0345	16.2501	15.8609	15.7198
PurelySDM, *λ* = 0.04, no fluid				
${\bar{\omega }}_{1}$	3.6621	1.609	1.1957	0.9937
${\bar{\omega }}_{2}$	23.2523	17.1006	16.6962	16.5498
local theory; fluid, *n*_k_ = 4				
${\bar{\omega }}_{1}$	1.9247	1.2982	1.0404	0.8923
${\bar{\omega }}_{2}$	2.4674	2.4674	2.4674	2.4674
${\bar{\omega }}_{3}$	12.1148	7.8824	7.2903	7.0561
${\bar{\omega }}_{4}$	22.2066	22.2066	22.2066	22.2066
PurelySDM, *λ* = 0.04; fluid, *n*_k_ = 4				
${\bar{\omega }}_{1}$	2.0206	1.3491	1.0781	0.9235
${\bar{\omega }}_{2}$	2.4723	2.4723	2.4723	2.4723
${\bar{\omega }}_{3}$	12.8574	8.3265	7.7124	7.4708
${\bar{\omega }}_{4}$	22.5977	22.5977	22.5977	22.5977

## Data Availability

Not applicable.
